# The effect of calcineurin inhibitors in the treatment of IgA nephropathy: A systematic review and meta-analysis (PRISMA)

**DOI:** 10.1097/MD.0000000000004731

**Published:** 2016-09-02

**Authors:** Wei Peng, Yi Tang, Zheng Jiang, Zi Li, Xuhua Mi, Wei Qin

**Affiliations:** a Division of Nephrology, Department of Medicine, West China School of Medicine; b West China Hospital, Sichuan University, Chengdu, Sichuan, China.

**Keywords:** calcineurin inhibitors, cyclosporine, immunoglobin anephropathy, meta-analysis, steroids, system review, tacrolimus

## Abstract

**Background:**

Immunoglobin A nephropathy (IgAN), the most prevalent form of primary glomerulonephritis, represents the leading cause of kidney failure among East Asian populations. Immunosuppressive treatment regimen, except for a 6-month trial of corticosteroids, has not been approved by the KDIGO guideline yet. Specific and effective treatment is still lacking. We decided to evaluate the efficacy and safety of the calcineurin inhibitors (CNIs) in the treatment of IgAN.

**Methods:**

Database from the Cochrane library, PubMed, Embase, CBM, CNKI, and CENTRAL databases were searched and reviewed up to March 2016. Literature was screened by 2 independent reviewers accordingly. Clinical trials were analyzed using Stata 12.0.

**Results:**

Five random control trials and 2 nonrandomized concurrent control trials were selected and included in this study according to our inclusion and exclusion criteria. The rates of complete remission in patients with IgAN were significantly increased in the group of CNIs (RR 1.56, *P* = 0.002). No statistical difference was observed in the rates of partial remission, or response between the CNIs and steroids alone. Additionally, CNIs resulted in a significant reduction in urinary protein (WMD 0.34, *P* = 0.002) and increase in serum albumin level (WMD 1.89, *P* = 0.013). No differences were found in the serum creatinine, estimated glomerular filtration rate, and rates of adverse effects including infection, hyperglycemia, and liver dysfunction.

**Conclusion:**

With present evidence, CNIs may be promising immunosuppressive agents for IgAN in future. However, large, long-term, multicenter trials are required to confirm our findings.

## 1 Introduction

Immunoglobin A nephropathy (IgAN) is pathologically characterized by the deposition of IgA immune complexes in the mesangium of the kidney.^[1]^ It is recognized to be the most prevalent form of primary glomerulonephritis worldwide, especially in China.^[2]^ The clinical manifestation is varied, of which microscopic hematuria and proteinuria are the most common ones. However, approximately 15% to 20% of affected patients progress to end-stage renal disease (ESRD) within 10 years, and 40% within 20 years.^[3]^ The therapeutic options are limited and remain controversial. Currently, the only recommendation from Kidney Disease Improving Global Outcomes (KDIGO) in IgAN based on unequivocal evidence is blood pressure control and the use of angiotensin-converting enzyme inhibitors (ACEIs) or angiotensin receptor blockers (ARBs).^[4]^ Proteinuria is regarded as the strongest predictor of unfavorable renal prognosis in IgAN. Therefore, its reduction is an important therapeutic goal in clinical practice.^[5]^ After 3 to 6 months of treatment with RAS inhibitors, a 6-month trial of corticosteroids is recommended if proteinuria remains persistent and >1 g per day. But patients are not always sensitive to corticosteroids, which leads us to take immunosuppressive agents into consideration.

As an autoimmune kidney disease, IgAN is reported to be responsive to some immunosuppressive therapy.^[6]^ Calcineurin inhibitors (CNIs) possess potent immunosuppressive properties. The suppression of the activation and proliferation of T cells by CNIs is due to the inhibition of the synthesis of interleukin (IL)-2, which leads to the suppression of secondary synthesis of various cytokines, such as IL-4, tumor necrosis factor-α.^[7]^ Although several studies indicated that CNIs, such as cyclosporine (CyA) and tacrolimus (TAC), are effective at treating IgAN, no systematic review has been performed on it. Therefore, this study sought to evaluate the efficacy and safety of these CNIs in comparison with corticosteroids in treating IgAN.

## 2 Methods

The data included are based on the studies published previously. Therefore, no ethical approval or patient consent is required.

### 2.1 Search strategy

A literature search was performed in PubMed, the Cochrane Library, the Embase databases, China Biology Medicine (CBM), and China National Knowledge Infrastructure (CNKI). PubMed (1966–February 2016), Embase (1974–February 2016), the Central Register of Controlled Trials (1999–January 2016), the Cochrane Renal Group (1999–January 2016), CBM, and CNKI were searched for the identification of relevant trials. The following search terms were used: IgA nephropathy, IgA nephritis, glomerulonephritis, IgA, Bergers disease, immunoglobulin A nephropathy, tacrolimus, TAC, FK506, prograf, cyclosporine A, CsA, CyA, Neoral, and calcineurin inhibitors. Relevant text words relating to eligible interventions were also searched. We also hand-searched the bibliographies of articles for additional references. The results were limited to human studies with no restrictions on language.

### 2.2 Inclusion criteria and risk of bias

Articles were selected and subsequently screened based on the patient problem intervention comparison outcome principle. Five random controlled trials (RCTs) and 2 nonrandomized concurrent control trials (NRCCTs) were finally selected. Study subjects were treated with corticosteroids alone or in combination with CNIs no less than 6 months, as KDIGO recommended a 6-month trial of corticosteroids. Only trials enrolling patients with biopsy-proven IgA nephropathy and clearly defined remission criteria, remission outcome data, and safety data were included. Full texts of all potential articles were retrieved and reviewed independently by at least 2 investigators. A risk of bias table recommended by the Cochrane risk of bias tool was used to assess the risk of bias of included trials.

### 2.3 Data extraction and management

Wei Peng and Yi Tang performed data extraction independently using standard data extraction forms, and Wei Qin was consulted when there was a discrepancy. For studies from which detailed data could not be extracted, the authors were contacted by email. Basic information such as first author, year of publication, study design, inclusion criteria, study sample size, basic characteristics of the study subject, intervention regimen, drug dosage, follow-up time, outcome data, and adverse effects was recorded for each study included.

### 2.4 Outcome measures

The primary outcomes were the complete remission (CR) rate, the partial remission (PR) rate, and the response rate, as defined by the sum of the complete and partial remission rates. The serum creatinine (sCr) level, estimated glomerular filtration rate (eGFR), serum albumin level, and 24-hour urine protein level were used as efficacy indexes, whereas the rates of infection, hyperglycemia, and liver damage were used as safety indexes. Complete and partial remission criteria and rates were established within each article and are described in Table 1. Proteinuria reduction was used as a criterion for remission and complete remission was defined as a reduction in proteinuria to less than 0.5 g/d in all studies. Some used even lower thresholds of less than 0.3 g/d, and several used serum creatinine levels and serum albumin levels as additional criteria for remission. Partial remission criteria varied between articles, but all required a greater than 50% or 25% reduction in proteinuria. When data were missing or incomplete, the investigators of the trials were contacted for clarification.

**Table 1:**
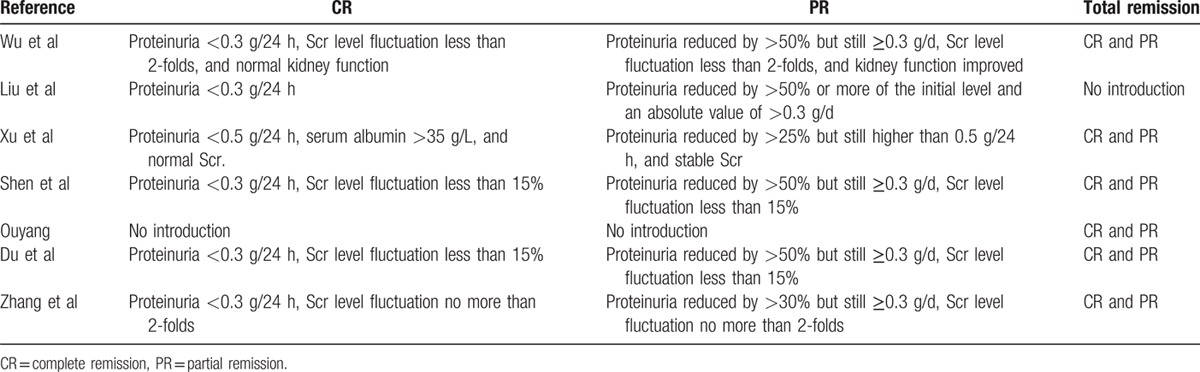
Definitions of clinical outcome in each study.

### 2.5 Statistical analysis

The meta-analysis was performed using STATA version 12.0 (STATA Corporation, College Station, TX). Relative risk (RR) and weighted mean difference (WMD) were chosen as effective sizes for dichotomous and continuous variables, which were described with a 95% confidence interval (CI). The *χ*^*2*^ test was used to analyze the heterogeneity of the trials. *P* >0.05 indicated that there was no statistically significant heterogeneity, therefore a fixed-effects model was applied; whereas *P* < 0.05 indicated statistically significant heterogeneity, therefore a random-effects model was applied. Publication bias was examined using a funnel plot. The symmetrical characteristics of the funnel plot were evaluated using the test proposed by Begg and Mazumdar where a symmetrical result demonstrated no publication bias, and asymmetry indicated potential publication bias.

## 3 Results

### 3.1 Study selection

**Figure 1:**
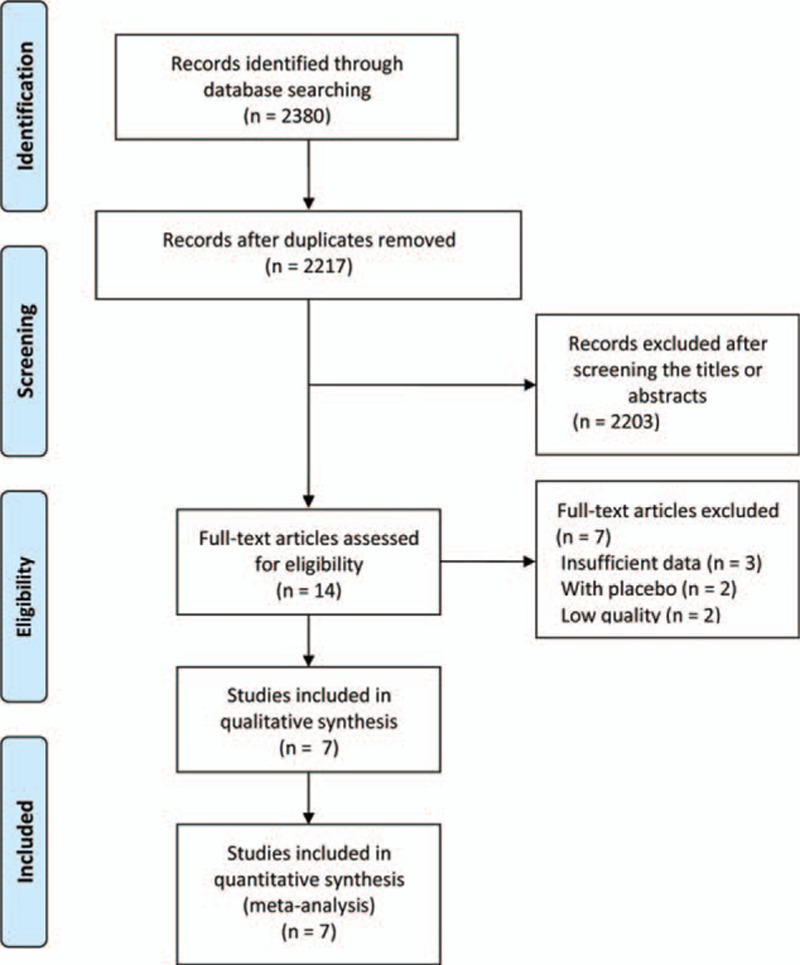
The flow chart of included studies in the meta-analysis.

We identified 2380 articles in the first search. Of these, after careful examination of the title and abstract, 2366 articles were excluded because 254 articles were duplicate references; 351 were conference abstracts; 913 were case reports, reviews and meta-analyses; 252 were basic researches, 92 were noncontrolled studies, and 504 were about posttransplant IgAN. Full texts of the remaining 14 articles were retrieved for further selection. An additional 7 articles were excluded, including 3 studies with insufficient data, 2 studies with a placebo, and 2 studies of low quality. Eventually, 7 studies were included in this systematic review and meta-analysis.^[8–14]^ The article search strategy used in our review is described in Fig. 1.

### 3.2 Trial characteristics

Characteristics of included studies are listed in Table 2, including 5 RCTs and 2 NRCCTs. In total, 370 patients were enrolled in the present meta-analysis with 184 patients in the CNIs treatment group and 186 in the steroid group. All patients had biopsy-proven IgA nephropathy. Risk of bias assessment included was performed using a risk of bias table recommended by Cochrane, as is shown in Table 3. Two studies^[9,12]^ have selection bias. None of these trials performed appropriate blinding method to avoid performance and detection bias.

**Table 2:**
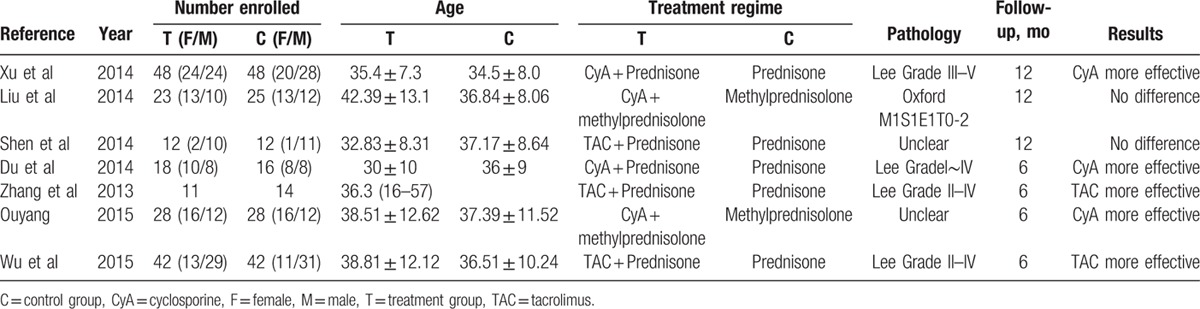
Characteristics of the studies included in this systematic review.

**Table 3:**
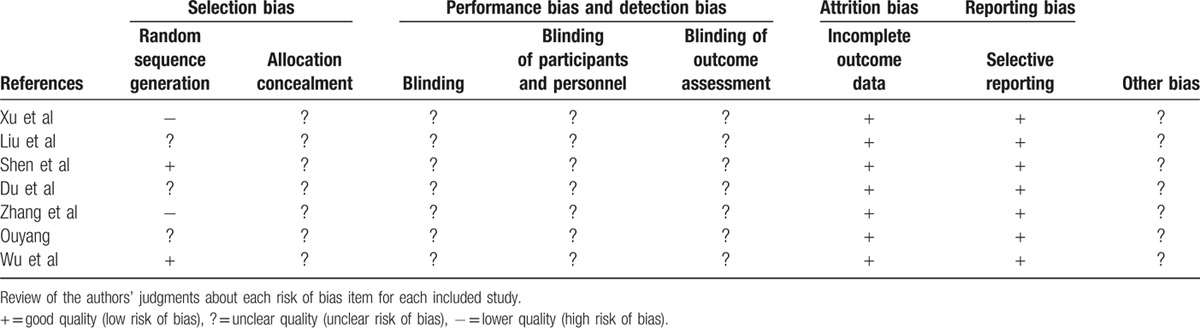
Risk of bias summary.

### 3.3 Efficacy and safety of the CNI in IgAN

**Figure 2:**
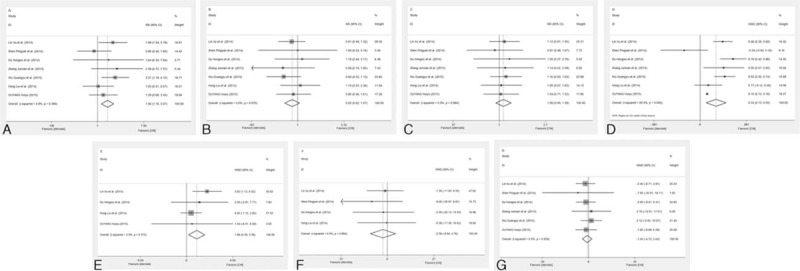
Meta-analysis on the therapeutic efficacy of the calcineurin inhibitor (CNI) and steroids. Comparison of complete response rate (A), partial response rate (B), response rate (C), proteinuria (D), serum albumin (E), the level of estimated glomerular filtration rate (F), and serum creatinine (G) between CNI and steroids treatment group.

In the present meta-analysis, the comparison of the efficacy and safety of the CNI and steroid in the therapy of IgAN patients included 7 trials (see Fig. 2). CNIs were orally administered for 6 to 12 months, and gradually tapered thereafter. Prednisolone (0.8–1.0 mg/kg/d) was administered for 4 to 8 weeks and subsequently tapered off within 1 year. Patients receiving CNIs demonstrated significantly increased CR rate (RR 1.56; 95% CI 1.18, 2.07; *P* = 0.002), as compared with the steroid therapy alone. However, No significant difference was observed in the PR rate (RR 0.82; 95% CI 0.62, 1.07; *P* = 0.15), or response rate (RR 1.09; 95% CI 0.93, 1.29; *P* = 0.28). Moreover, patients receiving CNIs (plus steroid) demonstrated a significant reduction in urinary protein excretion levels by the end of treatment, as compared with patients treated with steroid therapy exclusively (WMD 0.34; 95% CI, 0.13, 0.55; *P* = 0.002). Comparison of serum albumin level between CNIs and steroids treatment group included 4 trials, which showed a slightly higher albumin level in CNIs group (WMD 1.89; 95% CI 0.39, 3.39; *P* = 0.01). However, there were no significant differences in the eGFR (WMD −2.59; 95% CI −9.94, 4.76; *P* = 0.49) or serum creatinine levels (WMD −1.04, 95% CI −4.72, 2.64; *P* = 0.58) between CNIs and steroids treated patients.

### 3.4 Adverse effects of the CNI in IgAN

**Figure 3:**
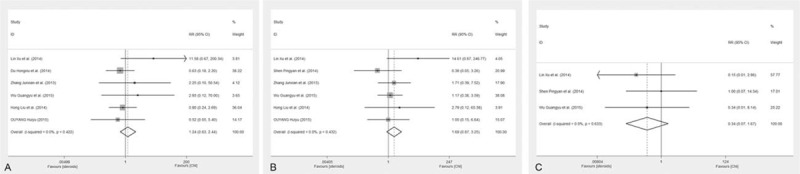
Meta-analysis on the adverse effects of the CNI and steroids. Comparison of the rates of infection (A), hyperglycemia (B), and liver dysfunction (C) between CNI and steroids treatment group.

Six studies were used to compare the adverse effects of the CNI and steroid during the therapy of IgAN patients (see Fig. 3). No differences were found in the rates of infection (RR 1.24; 95% CI 0.63, 2.44; *P* = 0.53) or hyperglycemia (RR 1.69; 95% CI 0.87, 3.25; *P* = 0.12). In addition, three studies reported the occurrence of liver dysfunction. But no significant difference was observed (RR 0.34; 95% CI 0.07, 1.67; *P* = 0.19).

### 3.5 Publication bias

**Figure 4:**
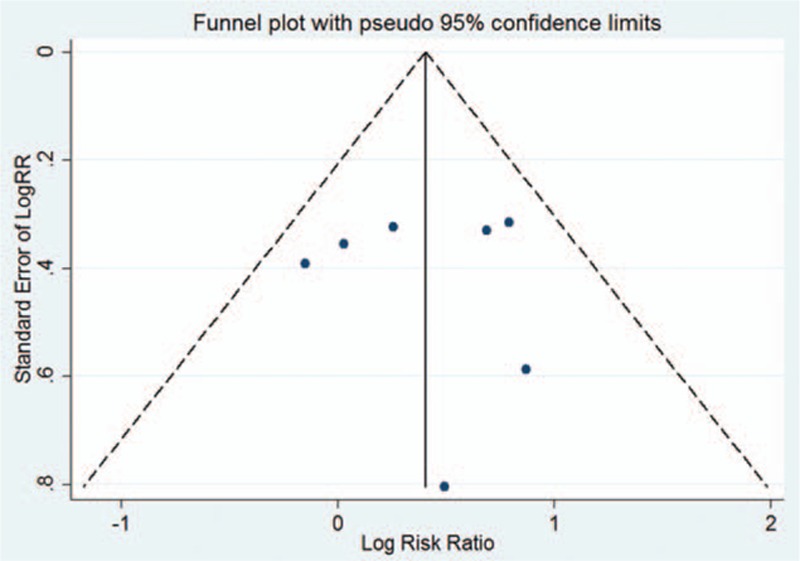
Funnel plot for the complete remission rate.

Publication bias was examined using funnel plots. The funnel plots of the 7 studies comparing the CNIs and steroids are shown in Fig. 4. The symmetric distribution suggests that there was no publication bias in these studies.

## 4 Discussion

IgAN, a kind of primary glomerularnephritis that was first identified by Berger and Hinglais in 1968, represents the leading cause of kidney failure among East Asian populations.^[15]^ Aberrant glycosylation of IgA1 elicits an autoimmune response, generating antiglycan antibodies.^[16]^ Consequent immune complexes deposit in the glomerular mesangium, which activates the complement pathway, stimulates mesangial cells, and induces secretion of cytokines, finally resulting in inflammation and fibrosis. Therefore, simply IgAN is an autoimmune disease wherein immune complexes induce renal injury. However, specific and effective treatment is still lacking. Only antihypertensive drugs, such as ACEI, ARB, turn out to be the useful intervention.^[17]^ However, antihypertensive medication is usually not applicable for the IgAN patients with mild to moderate proteinuria due to low blood pressure. Immunosuppressive treatment regimen for patients with IgAN, except for a 6-month trial of corticosteroids, has not been approved by the KDIGO guideline yet.^[18]^ Since IgAN is not always benign and still known as a leading cause of renal failure, it is urgent to develop a standard and reasonable immunosuppressive strategy for the patients with progressive IgAN. To date, CNIs perform well in many kidney diseases.^[19,20]^ It is reported that CNIs can reduce proteinuria effectively and rapidly in IgAN.^[21]^ To clarify the effect of the CNIs CyA and TAC on the treatment of IgAN, we performed this systematic review and meta-analysis.

In this study, 5 RCTs and 2 NRCCTs were included. Short-term parameters, including urinary protein, serum albumin, complete, and partial remission, were reviewed. Almost all the participants present with massive proteinuria >2 g daily. Complete proteinuria remission was usually defined as <0.3 g/d. A number of retrospective studies indicated that severity of proteinuria and low serum albumin are risk factors for kidney disease progression.^[22]^ Urinary proteins can induce tubulointerstitial damages, as is supported by many clinical researches and animal models.^[23,24]^ Serum albumin is widely recognized as a biomarker of nutritional status and inflammation. And it has been also identified during a long follow-up as an independent risk factor for renal outcomes in IgAN patients.^[25]^ In the present analysis, patients who received CNIs had significant reduction of proteinuria, and increase of plasma albumin concentration compared with steroids alone. Similar to our findings, several studies also indicated that patients with IgAN could experience significant improvement in proteinuria and hypoalbuminemia during CNI treatment,^[26]^ thus, leading to a high rate of complete clinical remission, as is also shown in our meta-analysis. Therefore, based on the results of current study, we think that CNI (CyA and TAC) could be an alternative treatment to IgAN patients with massive proteinuria when they refuse or are intolerant to steroids.

Traditionally, the immunosuppression mechanism of CNI involves inhibition of nuclear factor of activated T cells signaling in T cells.^[27]^ However, in recent years, it is widely accepted that CNI can stabilize the actin cytoskeleton in podocytes, ensure the viability of podocytes and maintain the integrity of the glomerular filtration barrier through variant mechanisms, thus, reducing proteinuria directly. Faul et al^[28]^ reported that inhibition of calcineurin in the kidney could maintain synaptopodin protein abundance in podocytes which is sufficient to safeguard against proteinuria. A recent study demonstrated that the antiproteinuric effect of CyA is mediated by regulating phosphorylation of an important actin nucleator, Wiskott–Aldrich syndrome protein-family protein 1, located in kidney glomerular podocytes.^[29]^ What is more, TAC may protect podocytes against injury by upregulating the expression of nephrin and podocin.^[30]^ The nonimmunologic actions distinguish CNIs from other immunosuppressive agents. Corticosteroid monotherapy is not beneficial for protecting kidney function.^[31]^ The addition of CNIs to corticosteroids can show a better therapeutic effect in patients with IgAN, particularly in those who do not respond to corticosteroid monotherapy.

Several large numbers of investigations and studies have reported various nephrotoxicity of CNIs.^[32,33]^ Deterioration of renal function during CNIs treatment, despite within-range trough drug levels, occurred in previous studies.^[21]^ Therefore, safety issues related to CNIs treatment have always been a concern in nephrologists and patients. In present study, no obvious nephrotoxicity directly related to CNIs was demonstrated. Tolerable adverse events including infection, hyperglycemia and liver dysfunction, showed a low incidence and no significant difference. What's more, serum creatimine and eGFR remained steady during the treatment, suggesting that CNIs would not compromise renal function of IgAN patients. However, the chronic nephrotoxicity is considered to be the Achilles’ heel of current immunosuppressive regimens and occurs at a median onset of 3 years.^[34]^ The data analyzed in the present study were obtained from short-term studies. In general, it is insufficient to draw a conclusion of the safety of CNI in treating IgAN. Future studies need long-term follow-up.

Unfortunately, the efficacy and safety of CNIs in the patients with IgAN in other ethnicities are unknown, and no relevant control trials were found. Based on the current study, CNIs demonstrate favorable effect for reducing proteinuria and maintaining renal function in Chinese patients with tolerable side effects. Accumulated evidence showed the significant impact of ethnic groups on the pharmacokinetics of CNIs and IgAN genetic risk.^[35]^ Multiethnic RCTs are needed to test the feasibility of CNIs in IgAN worldwide.

Our study had several limitations. The data analyzed in the present meta-analysis were obtained from short-term, small sample sizes, single-center studies. And we failed to obtain individual patient and original data, which may compromise our results. Additionally, our meta-analysis contained heterogeneity in pathological subtypes and drug dosages, and have an impact on the reliability of our results. Therefore, long-term, large-sample, multicenter RCTs are needed to confirm the efficacy and safety of CNIs in IgAN treatment.

## 5 Conclusion

In conclusion, our meta-analysis indicated that the short-term administration of CNIs in IgAN resulted in greater effect in the improvement of proteinuria and serum albumin with stable renal function and tolerable adverse side effects, as compared with steroids alone. Therefore, CNIs could be an alternative method of IgAN treatment especially for patients who are intolerant or unresponsive to steroids. Furthermore, large, high-quality multicenter trials are required to confirm our findings.

## Acknowledgment

We would like to warmly thank Dr Xiaoyuan Zou, a researcher working in Department of Epidemiology and Biostatistics, West China School of Public Health, Sichuan University, due to her help.
